# Evaluating current status of network pharmacology for herbal medicine focusing on identifying mechanisms and therapeutic effects

**DOI:** 10.1016/j.jare.2024.12.040

**Published:** 2024-12-25

**Authors:** Won-Yung Lee, Kwang-Il Park, Seon-Been Bak, Seungho Lee, Su-Jin Bae, Min-Jin Kim, Sun-Dong Park, Choon Ok Kim, Ji-Hwan Kim, Young Woo Kim, Chang-Eop Kim

**Affiliations:** a School of Korean Medicine, Wonkwang University, Iksan 54538, Republic of Korea; b Research Center of Traditional Korean Medicine, Wonkwang University, Iksan 54538, Republic of Korea; c School of Korean Medicine, Woosuk University, Jeonju 54986, Republic of Korea; d Department of Veterinary Medicine, Research Institute of Life Science, Gyeongsang National University, Jinju 52828, Republic of Korea; e School of Korean Medicine, Dongguk University, Gyeongju 38066, Republic of Korea; f Department of Nutritional Science and Food Management, Ewha Womans University, Seoul 03760, Republic of Korea; g Department of Clinical Pharmacology, Severance Hospital, Yonsei University Health System, Seoul 03722, Republic of Korea; h School of Korean Medicine, Pusan National University, Yangsan-si 50612, Republic of Korea; i School of Korean Medicine, Gachon University, Seongnam 13110, Republic of Korea

**Keywords:** Network pharmacology, Herbal medicine, Comprehensive evaluation, Therapeutic mechanisms

## Abstract

**Introduction:**

Network pharmacology has gained significant traction as a tool for identifying the mechanisms and therapeutic effects of herbal medicines. However, despite the usefulness of these approaches, their diversity underscores the critical need for a systematic evaluation to ensure consistency and reliability.

**Objectives:**

We aimed to evaluate the network pharmacological analyses, focusing on identifying the mechanisms and therapeutic effects of herbal medicines.

**Methods:**

We employed a comprehensive approach involving systematic data retrieval, network construction, and analysis. Herbal compounds and their targets were meticulously extracted from five distinct network pharmacology databases to ensure extensive coverage and high data reliability. Advanced network-based methods were used to identify key herbal targets and predict therapeutic effects, thereby enriching the depth and breadth of the analysis. Experimental validation was performed on prostate cancer models to substantiate the computational predictions.

**Results:**

The results of the recapitulating task for known herbal ingredient targets revealed distinct patterns in performance and coverage based on network construction and aggregation methods. We performed the same analysis to identify herbal targets and found that network centrality, path counts, and downweighted path counts had their own pros and cons. By comparing network-based methods, we found that considering the impact on the multiscale interactome yielded the highest accuracy in discriminating known therapeutic effects. Using optimal conditions, we successfully identified new indications for herbal medicines and validated these findings through follow-up *in vitro* and *in vivo* experiments.

**Conclusion:**

This study presents the first comprehensive and critical evaluation of the current network pharmacology analyses in the field of herbal medicine and provides valuable guidance for continued advances in the elucidation of the mechanisms and therapeutic effects.

## Highlights

• Essential mechanisms of herbal compounds are precisely identified and thoroughly analyzed.
• Rigorous evaluation of consistency across various network pharmacology databases is conducted.
• The choice of database is shown to significantly impact the understanding of known mechanisms.
• Advanced analytical techniques are demonstrated to be crucial for elucidating key mechanisms.
• The therapeutic potential of specific herbs in treating prostate cancer is validated *in vitro* and *in vivo.*

## Graphical abstract

**Figure:**
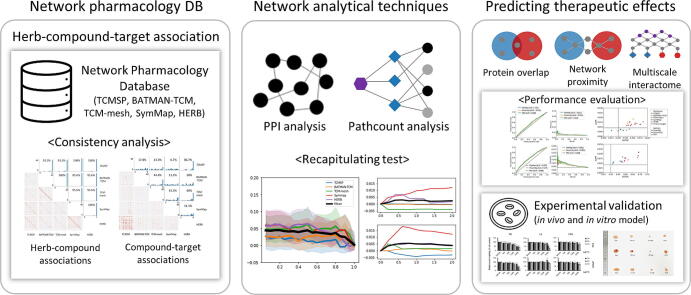

## Introduction

Herbal medicine, a common intervention in traditional Asian medicine, is used to treat a wide spectrum of diseases and pathological symptoms. Beyond its historical applications, it serves as a valuable source for novel drug discovery, demonstrating its potential in combating conditions such as COVID-19 and cancer [1], [2], [3], [4]. However, despite their rich history and potential, the application of herbal medicines is often based on empirical knowledge, and many of their mechanisms remain elusive. One of the primary reasons for this limitation is their complex nature, comprising multiple compounds that act on multiple targets [5], [6]. To address this complexity, researchers have adopted a network pharmacology approach that explores the relationship between drugs and diseases within the context of a biological network [7], [8]. Consequently, network pharmacology (NP), by elucidating intricate interactions and mechanisms, has become a pivotal tool in this field, witnessing exponential growth in research over the past decade.

Recent studies have sought to elucidate the mechanisms of action of herbal medicines using NP. Lee et al. delved into methodological trends in this analysis, identifying dominant databases (DBs) and observing their evolution over time [9]. Zhang et al. provided a comprehensive summary of the tools and DBs specifically employed for NP studies, including a comparison of the general statistics of herbal medicines across DBs [10]. Wang et al. showcased a method crafted by their research team for such analyses and detailed its progression over the years [11]. Despite these efforts, the practical utility of these analyses remains undetermined due to a lack of quantitative evaluation of the methods used for network pharmacological analyses. Without a standardized approach, the results from different studies can manifest significant variances, thereby causing inconsistencies in conclusions and potential misinterpretations in the application of herbal medicines. This highlights the pressing need for a systematic assessment of NP methods specific to herbal medicine research.

In this study, we systematically evaluated network pharmacological analyses of herbal medicines, focusing on their mechanisms and therapeutic effects. Here, we describe the characteristics of network construction methods across various DBs and their applications in deciphering their mechanisms and therapeutic effects. Subsequently, we evaluated the consistency of these network construction methods to assess the potential disparities in the representation of herbal ingredients and their target information across different DBs. Our analysis then explored the capacity to recapitulate the known targets of herbal medicines according to data sources and methods. We comprehensively assessed which network-based approaches were beneficial for classifying both the known and undiscovered therapeutic effects of herbal medicines. Finally, experimental validation was conducted on prostate cancer, which emerged as a prioritized result with therapeutic importance. This study sheds light on the current state of network pharmacological analysis techniques and their relevance to herbal medicine.

## Methods

### Herb-compound-target network construction

Data retrieval of herbal compounds and their corresponding target information was performed using five representative NP-DBs: TCMSP [12], TCM-mesh [13], BATMAN-TCM [14], SymMap [15], and HERB [16]. For TCMSP, TCM-mesh, and BATMAN-TCM, data were directly retrieved from each DB using the available resources provided. The HERB database provides both mining data from other databases and known therapeutic effect data. In this study, we used the mining data from HERB, which will be referred to as HERB (mining), while the known therapeutic effect data will be discussed in subsequent sections. For SymMap and HERB, compound information for herbs and target information for compounds were obtained using API calls in combination with Python's “requests” module based on a cross-reference ID table organized by individual entities.

An ID mapping process was then conducted on the assembled dataset to ensure consistency in the herb, compound, and target nomenclature across diverse data sources. Herbal names were mapped based on the cross-ID table provided by the HERB DB. This process was initially curated by the first author and further validated through a comprehensive review by the corresponding author, both of whom are licensed Korean medicine doctors with PhDs in herbal prescription. Subsequently, the compounds and targets were mapped to PubChem Compound IDs (CIDs) and Entrez Gene IDs using cross-reference ID tables from each database and the NCBI Gene DB, respectively. In the subsequent stages of analysis, only the data that were successfully mapped with these IDs (CIDs for compounds and Entrez Gene IDs for targets) were utilized. This strategy ensured the use of a consistent identification system across all data used in subsequent analyses.

### Multiscale interactome

The multiscale interactome is a network of interactions among proteins, between proteins and biological functions, and among biological functions themselves. The multiscale interactome used in this study was obtained from the data built by Ruiz et al. [17]. For protein–protein interactions (PPIs), they assembled 387,626 physical interactions between 17,660 proteins from seven major databases, including the Biological General Repository for Interaction Datasets, Database of Interacting Proteins, and Human Reference Protein Interactome Mapping Project. For protein-biological function interactions, they compiled 34,777 associations between 7,993 proteins and 6,387 biological functions from the Gene Ontology (human version). For biological-biological functional interactions, they constructed a hierarchy of biological functions consisting of 22,545 associations and 9,798 biological functions.

### Known targets and therapeutic effects of herbs and compounds

The known targets and therapeutic effects of herbs and compounds were identified using the HERB DB. The known targets are referred to as HERB (paper) in this study. This DB provides manually curated data on known targets and therapeutic effects associated with herbs and herbal compounds. During data extraction, we retrieved reference IDs from the HERB DB, which contained data related to known targets and therapeutic effects. The Python “requests” module was employed to retrieve references sequentially [18]. This allowed the collation of information pertaining to experimentally validated targets, denoted as 'paper-mined target genes' and 'paper-mined diseases'. The same procedure was performed to retrieve related proteins from the obtained disease list. After data extraction, our data comprised 2,403 compound-target interactions (CTIs) between 219 compounds and 875 targets, 256 herb-target interactions between 36 herbs and 182 targets, and 267,924 disease-protein associations between 320 diseases and 8,777 proteins.

### Evaluation of NP-DB consistency

The consistency among NP-DBs was evaluated using the recall metric. Recall is a measure designed to assess the uncommonness of the observed similarities. Specifically, for NP-DBs consistency analysis, a recall metric was used to evaluate the similarity of the same herbs or compounds across different DBs compared with the similarity within each DB [19]. This can be determined by calculating the proportion of similarity values in a reference distribution that is lower than the similarity value of the same object measured in another DB. The reference distribution is composed of Pearson's correlation distribution for all entities in the DB whose similarity to a particular object is to be measured. Pearson's correlation coefficient was calculated using one-hot encoding vectors to indicate the presence or absence of herbal compounds and their interactions with target compounds. High recall values correspond to higher-than-expected similarity values, providing an assessment of how well a particular pair of herbs or compounds matches relative to an appropriate null distribution.

### Identifying key targets of herbs

Network centrality, simple path count (PC), and degree-weighted pathcount (DWPC) are commonly used methods for identify key targets of herbs. Network centrality, also called topological analysis, refers to the calculation of each target's centrality in a subnetwork built based on PPIs between targets in a particular herb [20], [21]. Entities exhibiting high centrality are usually considered key targets. Network centrality measures typically include degree, betweenness, and closeness centralities. Degree centrality quantifies the number of direct connections maintained by a node within a network. Betweenness centrality estimates the extent to which a node functions as a bridge or broker in a network. Closeness centrality measures the proximity of a node to all other nodes within the network. Centrality measures were calculated using the following equations.

$$Degreecentrality\left(v\right)=deg\left(v\right)$$

where v denotes a specific node and deg(v) represents the number of edges connected to node v.

$$Betweennesscentrality\left(v\right)=\sum_{s\neq v\neq t}\frac{\sigma_{\mathrm{st}}\left(v\right)}{\sigma_{\mathrm{st}}},$$

where s and t refer to nodes in the network distinct from v, $\sigma_{\mathrm{st}}$ denotes the number of shortest paths from node s to node t, and $\sigma_{\mathrm{st}}\left(v\right)$ denotes the number of shortest paths from node s to node t that pass-through node v.

$$Closenesscentrality\left(v\right)=\frac{1}{\sum_{u}d\left(u,v\right)},$$

where u indicates a node in the network and d(u,v) signifies the shortest path distance from node v to node u.

PC refers to the number of paths of a specified metapath between the source and target nodes. However, PC does not adjust to the extent of graph connectivity along the path. Paths traversing high-degree nodes account for a large portion of PC, even though high-degree nodes often represent biologically broad or vague entities with limited informativeness. Consequently, relying solely on PC may lead to skewed interpretations, potentially overlooking specific and biologically relevant interactions owing to the dominance of high-degree nodes.

The DWPC algorithm mitigates the overrepresentation of high-degree nodes in PC calculations, thereby addressing the potential limitation of the PC method [22]. The DWPC algorithm originally downweights each path between a source and target node by calculating the path degree product (PDP) as follows:

1. Extracting all metaedge-specific degrees along the path ($D_{\mathrm{path}}$), where each edge contributing to the path adds two degrees.
2. Raise each degree to the negative w power, where w≥0 and serves as the damping exponent.
3. Multiplying all exponentiated degrees to generate the PDP.

In this method, we separated the damping parameter w into two distinct parameters, $w_{\mathrm{HC}}$ and $w_{\mathrm{CT}}$, which apply to degrees present in the herb-compound path and compound-target path, respectively. Consequently, when raising each degree to a negative power, we employed $w_{\mathrm{HC}}$ for degrees along the herb-compound path and $w_{\mathrm{CT}}$ for degrees along the compound-target path. The modified PDP and DWPC values were calculated as follows:

$$PDP\left(path\right)=\prod_{d_{\mathrm{HC}}\in D_{{\mathrm{path}}_{\mathrm{HC}}}}d_{\mathrm{HC}}^{{-w}_{\mathrm{HC}}}*\prod_{d_{\mathrm{CT}}\in D_{{\mathrm{path}}_{\mathrm{CT}}}}d_{\mathrm{CT}}^{{-w}_{\mathrm{CT}}},$$

where $D_{{\mathrm{path}}_{\mathrm{HC}}}$ and $D_{{\mathrm{path}}_{\mathrm{CT}}}$ refer to the paths between the herb-compound pair and compound-target pair, $d_{\mathrm{HC}}$ and $d_{\mathrm{CT}}$ denote the node degrees of $D_{{\mathrm{path}}_{\mathrm{HC}}}$ and $D_{{\mathrm{path}}_{\mathrm{CT}}}$, and $w_{\mathrm{HC}}$ and $w_{\mathrm{CT}}$ refer to the damping parameters of $D_{{\mathrm{path}}_{\mathrm{HC}}}$ and $D_{{\mathrm{path}}_{\mathrm{CT}}}$, respectively.

$$DWPC\left(h,\;t\right)=\sum_{\mathrm{path}\in Paths(h,t)}PDP\left(path\right),$$

where h refers to the herb, t denotes the target, and Paths(h,t) refer to the paths between h and t.

This modification allowed us to adjust the weighting of each path type individually, thereby providing a more precise representation of the network structure.

### Network-based methods for identifying therapeutic effects

Three network-based approaches were employed to predict the therapeutic effects of the herbal compounds and herbs: protein overlap, network proximity, and multiscale interactome. Protein overlap is based on the idea that ingredients with overlapping targets and disease-related proteins are likely to exert therapeutic effects. This can be calculated using the Jaccard Similarity between the set of drug targets *T* and the set of disease proteins *S*:

$$\frac{\left|T\cap S\right|}{\left|T\cup S\right|}.$$

Network proximity is based on the principle that the closer the targets of a compound are to disease proteins in a human PPI network, the more likely it is that the compound will affect the disease phenotype [23]. It is obtained by calculating the relative score of the shortest path length between the drug and disease based on the reference distribution. Let *T* be the set of drug targets and *S* be the set of disease proteins, where t ∈ *T* and s ∈ *S* represent the individual targets and proteins, respectively. d(s, t) denotes the shortest path length between nodes s and t in a network. Network proximity first computes the average closest distance, $d_{c}\left(s,t\right)$, between disease-associated proteins and ingredient targets as follows:

$$d_{c}\left(S,T\right)=\frac{1}{||T∥}\sum_{t\in T}^{\;}\min_{s\in S}d\left(s,t\right).$$

Next, a reference distance distribution is constructed using the values of $d_{c}\left(S,T\right)$ when *S* and *T* are randomly permuted into 1000 sets of proteins that match the size and degrees of the original disease proteins and targets in the network. Finally, the relative score is computed by taking the z-score of $d_{c}\left(s,t\right)$ with respect to the reference distribution:

$$Z_{d_{c}}=\frac{d_{c}-\mu_{d_{c}}\left(S,T\right)}{\sigma_{d_{c}}\left(S,T\right)},$$

where $\mu_{d_{c}}\left(S,T\right)$ and $\sigma_{d_{c}}\left(S,T\right)$ denote the mean and standard deviation of the reference distribution, respectively.

Multiscale interactome models compare the impact of drug treatment and disease perturbation on a network that integrates interactions between proteins and biological functions [17]. It predicts the disease association of drugs and natural products by calculating the diffusion profile $r\in R^{\left|V\right|}$ and then measures the correlation distance between them. A diffusion profile is computed through a matrix formulation with a power iteration as follows:

$$r^{\left(k+1\right)}=\left(1-\alpha \right)s+\alpha \left(r^{\left(k\right)}M+s\sum_{j\in J}r_{j}^{\left(k\right)}\right),$$

where $r^{\left(k\right)}$ denotes the diffusion profiles at k-th state, α denotes the probability of the walker continuing its walk at a given step rather than restarting, $s\in R^{\left|V\right|}$ denotes a restart vector that sets the probability that the walker will jump to each node after a restart, and M denotes a biased transition matrix derived from a directed multiscale interactome and set of scalar weights that encode the relative likelihood.

These procedures are repeated until the convergence of the power iteration computation is as follows:

$$\|r^{\left(k+1\right)}-r^{\left(k\right)}|{|}_{1}>\varepsilon ,$$

where ε denotes the tolerance parameter and was set to $1\times 10^{-6}$, which is the same value as that in the previous study [17].

The correlation distance of drug and disease diffusion profile is calculated as follows:

$$1-\frac{\left(r^{\left(c\right)}-{\overset{¯}{r}}^{\left(c\right)}\right)∙\left(r^{\left(d\right)}-{\overset{¯}{r}}^{\left(d\right)}\right)}{\|{(r}^{\left(c\right)}-{\overset{¯}{r}}^{\left(c\right)})|{|}_{2}\left\|\left(r^{\left(d\right)}-{\overset{¯}{r}}^{\left(d\right)}\right)\right|{|}_{2}},$$

where r(c) and r(d) denote the diffusion profiles of the drug and disease, respectively.

### Performance measure

Performance evaluation was conducted under two main scenarios within our study. First, we assessed whether the predicted targets of herbs or compounds derived from network construction and analyses corresponded to the known targets. Second, it was used to evaluate the predictive performance of known therapeutic effects based on the targets of herbs or compounds using various methods.

The choice of the evaluation metric depended on the nature of the predicted outcomes. For binary predictions (i.e., the interaction between targets and herbs/compounds or predictions based on the percentile threshold of network centrality or PC), we used metrics such as the Matthews Correlation Coefficient (MCC), precision, coverage, and recall. These values were calculated using the following formulae:

$$\mathrm{MCC}=\frac{\left(TP\times TN-FP\times FN\right)}{\sqrt{\left(TP+FP)\times (TP+FN)\times (TN+FP)\times (TN+FN\right)}},$$

$$\mathrm{Precision}=\frac{\mathrm{TP}}{\mathrm{TP}+FP},$$

$$\mathrm{Coverage}=\frac{\mathrm{TP}}{\mathrm{TP}+FN},$$

$$\mathrm{Recall}=\frac{\mathrm{TP}}{\mathrm{TP}+FN}$$

where TP represents true positives, TN represents true negatives, FP represents false positives, and FN represents false negatives.

For continuous predictions (i.e., predictions about known herbs based on DWPC or prediction scores for the therapeutic effects of known herbs/compounds), performance was evaluated using the Area Under the Receiver Operating Characteristic curve (AUROC) and Area Under the Precision-Recall curve (AUPR). These metrics provide an overall measure of prediction performance, balancing the trade-off between sensitivity and specificity for AUROC and precision and recall for AUPR.

### Chemicals and reagents

Anti-Bcl-2, anti-Bax, anti-Cdk2, anti-Cdk6, anti-p19, anti-p21, and anti-p27 antibodies were purchased from Santa Cruz Biotechnology (Dallas, TX). Anti-Mcl-1, anti-caspase-3, anti-PARP, anti-STAT3, anti-Cyclin D1, and anti-β-actin were purchased from Cell Signaling Technology (Danvers, MA). HRP-conjugated anti-rabbit IgG and anti-mouse IgG antibodies were acquired from Enzo Life Sciences (Farmingdale, NY). 3-(4,5-dimethylthiazol-2-yl)-2,5-diphenyl-tetrazolium bromide (MTT) and crystal violet solution were purchased from Sigma-Aldrich (St. Louis, MO). Annexin V/propidium iodide was purchased from BD Bioscience (San Jose, CA). Puerariae Radix (PR), Lithospermum Erythrorhizon (LE), and Granati Fructus (GF) are medicinal herbs certified by the Korea FDA and were sourced from an hGMP facility (Nonglim Saengyak, Seoul, KOREA). The extracts of these herbs were prepared according to previously described methods [24].

### Cell lines and cell culture

PC3 and LNCap cells (obtained from the American Type Culture Collection, Rockville, MD) were cultured in RPMI 1640 medium supplemented with 10% fetal bovine serum, 50 μg/mL streptomycin, and 50 units/mL penicillin. The cells were cultured at 37 °C with 5% CO_2_ in a humidified atmosphere.

### Animal housing and xenograft experiments

Six-week-old male BALB/c nude mice (18–20 g) were purchased from Samtako Inc. (Osan, Republic of Korea). Mice were housed at 22 ± 3 °C and 54% ± 5% relative humidity conditions in a 12/12h light and dark-controlled room (light: 7:00–19:00*h*, dark: 19:00–7:00*h*). The mice were provided ad libitum access to rodent food and water. All experimental animal procedures were approved by the Gyeongsang National University Institutional Animal Care and Use Committee (GNU-240228-M0048) and conducted in accordance with the guidelines of the National Institutes of Health (NIH publication).

PC3 cells were grown to 80% confluence and harvested for xenograft experiments. The cells were resuspended in phosphate-buffered saline at a concentration of 2 × 10^7^ cells/ml. Each mouse, aged 7 weeks, was subcutaneously injected with 1 mL of the cell suspension on the left side. The mice were grouped and allowed for the tumors to grow to a target volume of 200 mm^3^. Upon reaching 200 mm^3^ tumor volume, the drug group was orally administered PR, LE, and GF at doses of 500 and 1500 mg/kg/body weight three times a week for 15 days. Each control group was orally administered with (vehicle) and the positive control group was intraperitoneally injected with 8 mg/kg/day doxorubicin three times a week. Tumor growth and inhibition were measured daily using digital calipers. Tumor volume was calculated using the following formula: tumor volume (mm^3^) = tumor diameter × short diameter2/2. At the end of the animal experiment (15 days), the mice were sacrificed, and the tumors were dissected.

### Cell proliferation/viability assay

PC3 and LNCaP cells were cultured in a 48-well plate at a density of 5 × 10^3^ cells per well for 24h. Subsequently, PR, LE, and FPG were each treated at various concentrations for 24, 48, and 72h. Following the incubation period, MTT treatment was conducted for 2 h at 37 °C. After incubation, the medium was removed, and 300 μL of DMSO was added to each well to dissolve the formazan crystals. Absorbance was determined at 570 nm using an ELISA microplate reader (Agilent Technologies, Santa Clara, California).

### Clonogenic assay

PC3 and LNCaP cells were plated in a 6-well plate at a density of 1 × 10^3^ cells per well and treated with PR, LE, or FPG for 24h. Subsequently, the medium was replaced with drug-free medium, and the cells were cultured for an additional 8 days. After an 8-day incubation period, the medium was suctioned, and the cells were fixed with 4% formalin. The fixed cells were stained with 1% crystal violet solution and air-dried at room temperature.

### Flow cytometry

PC3 and LNCaP cells were plated in a 6-well plate at a density of 1 × 10^5^ cells per well for 24h. Subsequently, PR, LE, and FPG were administered to the cells at specified concentrations for 72h, followed by cell harvesting using trypsin. The cells were stained with Annexin V/propidium iodide (BD Biosciences, San Jose, CA) according to the manufacturer's instructions. The stained cells were assessed using an Accuri C6 flow cytometer (Accuri Cytometers Inc., Ann Arbor, MI) and 10,000 events were recorded.

### Immunoblot analysis

PC3 and LNCaP cells were plated in a 60Ø dish at a concentration of 3 × 10^5^ cells. Subsequently, PR, LE, and FPG were administered at specified concentrations for 72h. Cell lysates were prepared using RIPA buffer at 4 °C, and protein quantification of the lysates was performed using a BCA assay kit (Thermo Fisher Scientific Inc., Waltham, MA). The quantified proteins were separated by sodium dodecyl sulfate–polyacrylamide gel electrophoresis and subsequently transferred onto a PVDF membrane. After sequentially attaching primary and secondary antibodies to the transferred membrane, chemiluminescent signals were captured using a ChemiDoc image analyzer (Vilber Lourmat, France).

### Scratch wound healing assay

PC3 and LNCaP cells were incubated in a 24-well plate until they reached approximately 90% confluency. Subsequently, a wound (a mono line) was created by scratching the surface using a 200 μL pipette tip. The process of co-culturing cells with PR, LE, or FPG at specified concentrations for 72h was observed using an automated microscope (BioTek Lionheart, Winooski, VT).

### Statistical analysis

Statistical analyses of *in vitro* and *in vivo* experiments were performed by checking the normal distribution of the data and considering the number of groups under investigation. For two-sample evaluations, normality was assessed using the Shapiro–Wilk test. Subsequently, a two-tailed Student’s *t*-test was conducted for pairwise comparison of groups exhibiting a normal distribution, whereas the Mann–Whitney *U* test was used when the normality assumption was not met. Similarly, for comparisons involving multiple groups, normality was first examined using the Shapiro–Wilk test. Datasets conforming to a normal distribution were then analyzed using one-way ANOVA coupled with Dunnett’s test; in instances where normality was not observed, the Kruskal–Wallis ANOVA with Dunn’s post hoc test was applied. In cases where the effects of different treatments and their interactions were evaluated, two-way ANOVA was employed. This method accounted for the variability between different treatment groups and the interaction effects of combined treatments. The criteria for establishing statistical significance were set at *p* < 0.05 or *p* < 0.01.

## Results

### Overview of network construction and analysis

NP-DBs are primary resources for constructing networks of herbal medicines. They offer comprehensive information about the compounds and targets of herbal medicines, enabling researchers to build networks of specific herbal medicines. In this section, we summarize the network construction and analysis methods provided by representative NP-DBs: TCMSP, BATMAN-TCM, TCM-Mesh, SymMap, and HERB (Table 1. These DBs facilitate NP analysis of individual herbs, which many researchers have utilized and cited in their studies. These DBs also provide raw files or APIs, facilitating large-scale analyses.

**Table 1:** Description, statistics, and methods of representative databases that provide network pharmacological analysis for herbal medicines. H-C: herb-compound associations, C-T: compound-target associations. *Prediction method.

Name (published year)	Description	Statistics	Methods		Website (PMID)
			H-C	C-T	
TCMSP (2014)	A system pharmacology platform that provides information on ingredients, targets, and diseases of herbal medicines.	499 herbs, 29,384 ingredients, 3,311 targets	Literature search	Drugbank, HIT, and SysDT*	tcmsp-e.com/tcmsp.php (24735618)
BATMAN-TCM (2016)	A bioinformatics analysis tool for molecular mechanisms of TCM	8,159 herbs, 25,210 ingredients, 14,298 targets	TCMID	DrugBank, KEGG, TTD, and Similarity score*	bionet.ncpsb.org.cn/batman-tcm (26879404)
TCM-mesh (2017)	A system for providing network pharmacological analysis for TCM	6,235 herbs, 383,840 ingredients, 14,298 targets	TCMID	STITCH	bionet.ncpsb.org.cn/batman-tcm (28588237)
SymMap (2019)	An integrative database of TCM enhanced by symptom mapping	698 herbs, 25,975 ingredients, 20,965 targets	TCMID, TCMSP, and TCM-ID	HIT, TCMSP, HPO, DrugBank, and NCBI databases	symmap.org (30380087)
HERB (2021)	A high-throughput experiment- and reference-guided database of TCM	7,263 herbs, 28,212 ingredients, 12,933 targets	SymMap, TCMID 2.0, TCMSP 2.3, and TCM-ID et al.	SymMap, HIT, TCMSP, and TCMID	herb.ac.cn (33264402)

Network pharmacological analysis of herbal medicines usually begins with the construction of herb-compound networks. Most NP-DBs contain ingredient information from databases such as TCMID [25] and TCM-ID (Table 1). NP-DBs typically assign a unique ID to each compound and provide the corresponding PubChem CID. This aids researchers in identifying the properties of compounds and mapping additional information for subsequent analyses. Notably, TCMSP offers data on drug-likeness (DL) and predicted oral bioavailability (OB) of compounds, enhancing the ability to screen potential bioactive compounds [26]. Approximately one-third of the pertinent studies employed compounds surpassing the suggested thresholds (i.e., drug-likeness and oral bioavailability above 0.18 and 0.3, respectively) for their analyses [27], [28]. These thresholds were used to maximize bioactive compound extraction while maintaining a manageable number of candidates [29]. This information was integrated into SymMap and HERB for a comprehensive overview of herbal medicine bioavailability.

**Fig. 1:**
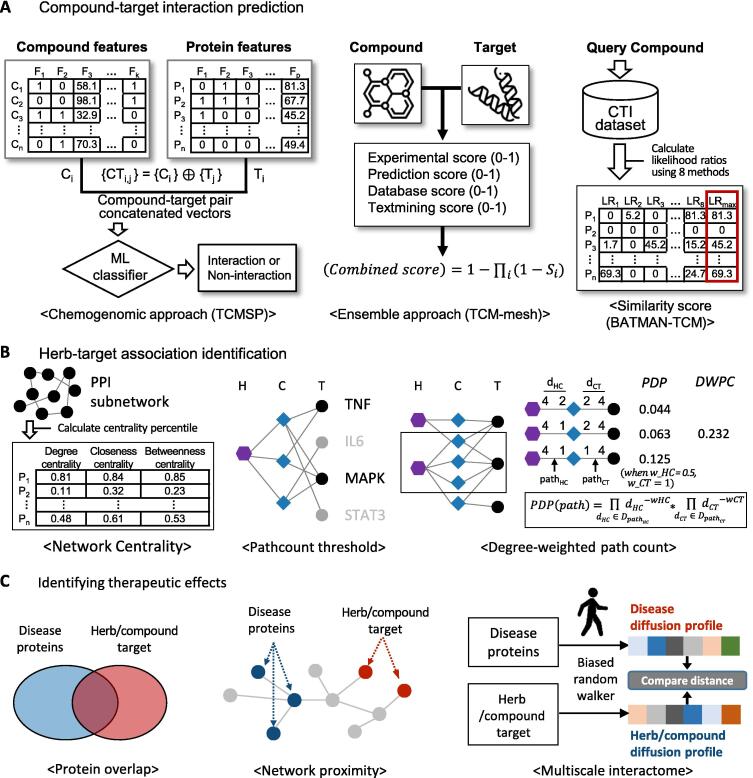
**Analytical techniques utilized in network pharmacology analysis. (A)** Compound-target prediction methods in network pharmacology database. Chemogenomic approach predicts interactions using vectors formed by concatenating the features of compounds and their targets. Ensemble approach relies on a combined score computed by ensembling scores derived from heterogeneous sources. Similarity score utilizes the maximum likelihood ratio based on various similarity scores for query compound-target pairs. (**B**) Herb-target association identification. Network centrality identifies key targets within the target network by evaluating node percentiles for degree, closeness, and/or betweenness and selecting those surpassing a specific threshold. Pathcount threshold designates key targets based on the presence of a specified or greater number of paths from an herb to that target. Degree-weighted path count downweights the pathcount for compounds found in many herbs (herb-compound degree, d*_HC_*) and compounds with multiple targets (compound-target degree, d*_CT_*). The example on the right panel illustrates the computation of DWPC values for herb-target pairs enclosed within the box, using parameters *w_Hc_*= 0.5 and *w_CT_*= 1. (**C**) Compound-disease prediction methods. Protein overlap calculates the overlapping targets between herb targets and disease-related targets. Network proximity calculates the relative distance between herb targets and disease-related targets in protein–protein networks. Multiscale interactome measures the influence of disease or herb/compound targets on the multiscale interactome and then calculates the distance between them.

The subsequent phase of network construction involved identifying the targets of compounds found in the herbal medicines (Fig. 1A). The sources of target information for herbal compounds can be broadly categorized into two primary types: experimentally validated and predicted. Databases such as the Herbal Ingredient Target [30], [31] and DrugBank [32] are typically employed to gather experimentally validated targets; they house information curated manually from the literature based on *in vitro* or biochemical experimental results. However, a potential limitation of this approach is the sparse nature of data on these experimentally validated targets. Relying solely on these targets limits the scope of herbal medicine analysis. Thus, NP-DBs provide predictive target information for herbal medicine components using various methods. For instance, Yu et al. proposed the SysDT model [33] to forecast CTIs using both known and unknown compound-target associations alongside protein and compound descriptors. TCMSP uses this model to predict the targets of Chinese medicinal ingredients. Liu et al. introduced a CTI prediction technique based on compound and target similarity scores [34]. BATMAN-TCM employs this method to identify the targets of herbal medicinal ingredients. TCM-Mesh curates the target data of herbal medicine components by incorporating information from STITCH [35], which aggregates scores from various sources, including experiments, predictions, DB consultations, and text mining.

The constructed network was analyzed to uncover the key mechanisms of action of the herbs. To identify key targets, researchers often construct protein–protein interaction (PPI) subnetworks by mapping herbal medicine targets onto the global PPI network (Fig. 1B). Within these target-specific PPI subnetworks, network centrality analysis is performed to identify hub nodes that play central and significant roles in the network structure, assuming these are key targets of herbal medicines [36]. Typically, researchers measure various properties such as degree centrality, closeness centrality, and betweenness centrality, which indicate the importance of nodes in a network. Targets exhibiting centrality values above certain thresholds are usually considered key targets. PC analysis, similar to degree analysis, assumes that targets with overlapping interactions with ingredients are likely to be key targets [37], [38]. This analysis allows for the consideration of the cumulative effects of herbal compounds on the targets. It computes the number of PCs from the herbs to the targets and selects those where the number exceeds a certain threshold, such as the median value. However, a potential limitation is treating all path counts (PCs) equally, regardless of whether they originate from common ingredients or those with numerous targets. High-degree nodes often represent biologically general or nonspecific entities, offering limited specific information [22]. Therefore, downweighting PCs associated with high-degree nodes can refine the analysis, potentially leading to a more accurate understanding of key mechanisms.

Network-based approaches also play a crucial role in leveraging the identified herbal targets to identify the therapeutic effects of herbal medicines. The principal or promising approaches among the network-based methods include protein overlap, network proximity, and multiscale interactome approaches (Fig. 1C). Protein overlap is based on the hypothesis that herbal medicines that share targets in specific diseases exhibit therapeutic efficacy [39]. Network proximity hypothesizes that herbal medicines with targets close to the disease proteins in the network exert therapeutic effects [23]. Network proximity employs z-scores to quantify the relative closeness of herbal targets to diseased proteins by comparing the observed distances with the distribution of distances from randomly selected proteins. The multiscale interactome refers to a network consisting of physical interactions between proteins and a hierarchy of biological functions [17]. One study found that simulating the propagation of a compound or disease on a multiscale interactome demonstrated state-of-the-art performance in predicting therapeutic effects and uncovered key mechanisms. Using this framework, Bak et al. successfully identified the active compounds in *Bupleuri Radix* and their key mechanisms against oxidative liver injury [40].

### Consistency analysis across NP-DBs

**Fig. 2:**
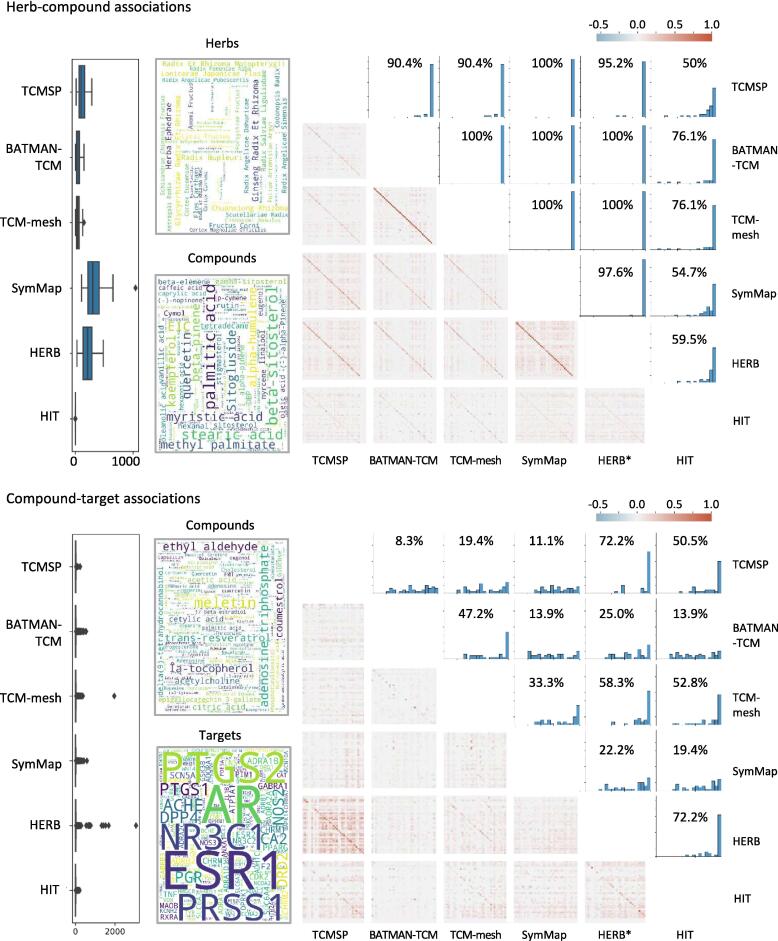
**Characteristics and consistency of information across various network pharmacology databases.** Herb-compound (upper figure) and compound-target (lower figure) characteristics by database. In each subfigure: the left panel displays the distribution of associations by database; the central panel highlights frequently occurring entities within the associations; and the right panel shows a correlation heatmap of herbs or ingredients between database pairs (lower triangle), paired with a distribution plot for recall percentiles (upper triangle). Within each correlation heatmap, the diagonal line represents the correlation values for the ingredients of identical herbs and the target information of the ingredients between the database pairs. Each distribution plot indicates the proportion of samples that achieved a recall value of 95% or higher.

We explored the characteristics and discrepancies of the ingredients and target information within the NP-DBs. We first measured the MCC values between known targets and those available in each DB, building on the herb and herbal ingredient analyses described in the previous section. Our analysis focused on 42 commonly used herbs in the East Asian region, each containing at least three ingredients, along with 36 herbal ingredients for which target information is available in all NP-DBs. This selection criterion aimed to analyze herbal medicines commonly used in practice, addressing issues related to their practical utilization. We compared the number of ingredients per herb across selected DBs and found that the average number of ingredients per herb was 129.6 (Fig. 2A). Specifically, SymMap and HERB had higher counts, whereas BATMAN-TCM, TCM-Mesh, and HIT had fewer ingredients. We also identified 80 common ingredients across all DBs, whereas 903 ingredients were unique to specific DBs (Supplementary Fig. 1). To assess the impact of these frequency discrepancies, we performed consistency analysis by calculating the relative ranking of correlations for the same herb across different DBs. This analysis presupposes that if ingredient information consistently appears across DBs for a specific herb, there should be a higher correlation in the ingredient information for that herb than for different herbs in separate DBs. We found that, notably, the only exceptions were all pairs involving the HIT DB; for all other pairs, the recall rate exceeded 90% in 10 out of 15 DB pairs. This suggests that the differences in herbal ingredient information across DBs may have a limited impact.

We further explored whether a similar pattern existed in the target information for the selected ingredients. We counted the number of targets in the ingredients and found that even for the same ingredient groups, the number of targets varied significantly between DBs (Fig. 2B). Although only 189 targets were consistently identified across all DBs, specific DBs offered up to 1,662 unique targets (Supplementary Fig. 2). For quantitative assessment, we performed a consistency analysis by computing the relative rankings of correlations between ingredients across pairs of DBs. Notably, we observed low correlation values for the same ingredients across different DB pairs. Our recall analysis showed that, even when performing the same analysis, none of the DB pairs had a recall rate exceeding 90%. This indicates that the target information for the same ingredient can diverge, depending on the DB used for the analysis. Our findings highlight the urgent need for a comprehensive, large-scale analysis of NP-DBs.

### Impact of NP-DBs on recapitulating known mechanisms

To evaluate the effectiveness of the target information provided by the NP-DBs, we conducted a recapitulation task focused on the known targets of herbs and their ingredients. Therefore, we grouped all ingredient-target and herb-target associations into known and unknown associations. Notably, the density of known associations was low (1.3% for ingredient-target and 3.8% for herb-target), and unknown associations also contained potentially positive samples. Although these conditions pose obstacles to accurate performance evaluation, they still provide valuable insights into the characteristics of the information source and analysis method.

**Fig. 3:**
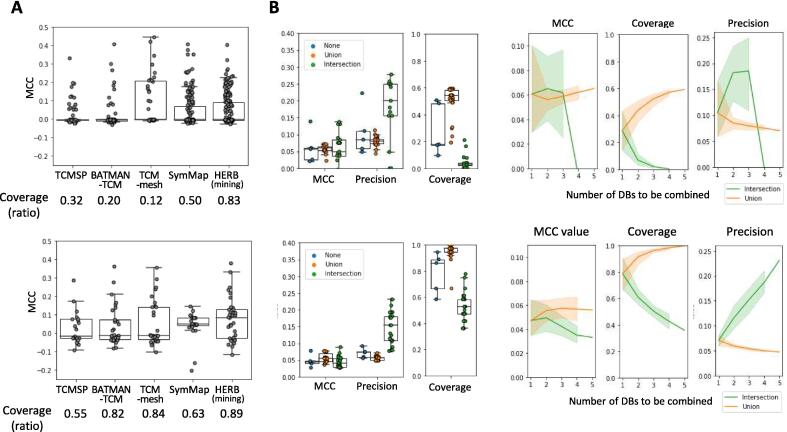
**Recapitulation performance for herbal compounds (upper figure) and herbs (lower figure) across different databases and combinations. (A)** Distribution of recapitulating performance and coverage for known targets in compounds and herbs by database. **(B)** Distribution of performance in rediscovering known targets by platform combination methods and the number of platforms used in the combination.

We first measured MCC values between known targets and those available in each DB, based on the herb and herbal ingredient analyses described in the previous section. All DBs outperformed the chance level, with the TCM-Mesh achieving the highest score of 0.14 (Fig. 3A, upper panel). We also measured the coverage of each DB to predict ingredients with known targets. On average, the DBs achieved a coverage of 0.40, with HERB and SymMap notably standing out at 0.83 and 0.50, respectively. These findings indicated that the reliability and coverage of herbal compounds exhibited unique patterns across different DBs.

We further analyzed the changes in recapitulation performance based on the combinations and aggregation methods across the NP-DBs (Fig. 3B, upper panel). Initially, we found that intersecting the target information from TCM-Mesh and HERB yielded the highest precision score (0.278). Among combinations with coverage greater than 0.5, uniting data from TCMSP, TCM-Mesh, SymMap, and HERB showed the best performance, with an MCC of 0.07. We observed minimal changes in performance when comparing union and intersection methods for aggregating target information, with all cases having an MCC of 0.06. However, when using the intersection method, we observed increased precision at the expense of reduced coverage, whereas the union approach yielded the opposite result. As the number of combined platforms increased, we observed a more pronounced tradeoff relationship between the MCC value and coverage.

We extended the analysis to evaluate the performance of each NP-DB and their combinations in recapitulating known target information at the herb level. Our results demonstrated that all DBs maintained coverage exceeding half, while achieving a performance that surpassed chance level (Fig. 3A, lower panel). Specifically, the highest coverage and MCC values were observed when HERB was used. In contrast, TCMSP displayed the lowest values for both metrics. Like our findings at the ingredient level, we observed a tradeoff between precision and recall based on the aggregation method used for NP-DBs at the herb level (Fig. 3B, lower panel). Overall, our results highlighted that the methods and combination strategies employed to construct networks for herbal medicines can significantly influence both predictive performance and coverage.

### Influence of analytical techniques on recapitulating known mechanisms

We then assessed how network analysis techniques influenced the recapitulation performance of known mechanisms in herbal targets. Initially, we explored whether PPI network analysis of the herbal targets could help elucidating these mechanisms. Subnetworks were constructed with targets from each DB as nodes and known PPIs forming the edges. Subsequently, three types of network centrality (degree, betweenness, and closeness) were measured for each node. We then evaluated the recapitulating performance while varying the percentile thresholds for network centrality, classifying targets as “known” when they exceeded the threshold, and “unknown” otherwise. The results indicated that as the centrality score increased, the MCC values generally decreased across most DBs. This trend was particularly evident for BATMAN-TCM, TCM-Mesh, and HERB. However, SymMap and TCMSP showed tendencies for increased MCC values within certain centrality thresholds (0.4–0.8). Our findings indicate that the benefits of using network centrality analysis in target protein networks vary depending on the specific conditions and DB, suggesting that this approach may not always be the most effective for identifying core herbal targets.

**Fig. 4:**
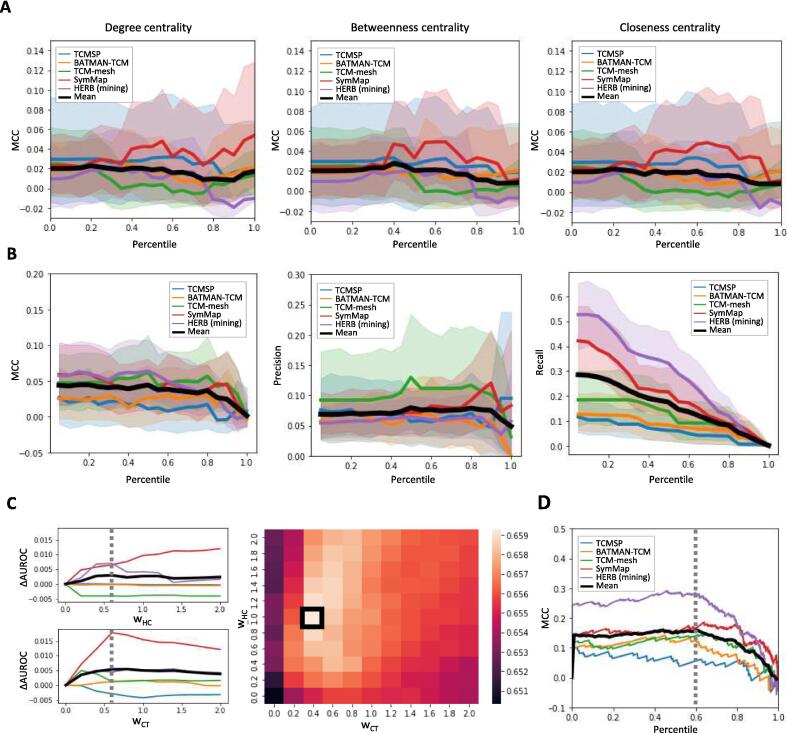
**Performance distribution of herb-target associations based on network analysis methods. (A**) Performance changes based on PPI network analysis of herb targets. Each plot represents the Matthews correlation coefficient value for herb-target pairs exceeding a given centrality percentile threshold for known herb targets. **(B**) Performance distribution according to path count percentile. Each plot displays the distribution of the Matthews correlation coefficient, precision, and recall between herb-target pairs exceeding a specified path count percentile threshold and known herb-target pairs. **(C)** Performance variation based on downweighting of the herb-compound-target network. The left plot depicts the AUROC value between the path count and known herb-target pairs as wHC and wCT vary. The right heatmap illustrates the average AUROC values for potential combinations of the downweighting parameters. **(D**) Performance variation according to downweighted path count percentile based on optimal conditions.

Next, we focused on variations in performance for recapitulating herbal targets based on path count (PC) percentile thresholds. Initially, we observed a continuous decline in the MCC value starting from a PC threshold of 0.5 (Fig. 4B). However, precision exhibited a slight increase within the 0.5–0.8 threshold range, while recall consistently decreased. Furthermore, we investigated how downweighting PCs, particularly those involving high-degree nodes, influenced the elucidation of key herbal targets. Using the DWPC algorithm, we performed the same analysis across a wide range of thresholds for the weight of the herb-ingredient path ($w_{\mathrm{HC}}$) and ingredient-target path ($w_{\mathrm{CT}}$). We found that, as both the $w_{\mathrm{HC}}$ and $w_{\mathrm{CT}}$ thresholds increased, the AUROC values also generally increased (Fig. 4C). Specifically, our findings indicated that, except for the TCM-Mesh for $w_{\mathrm{HC}}$ and TCMSP for $w_{\mathrm{CT}}$, employing the DWPC algorithm to downweight PCs generally contributed to performance enhancement across most DBs (Supplementary Fig. 3). By exploring various combinations of weighting parameters, we determined the optimal performance when $w_{\mathrm{HC}}$ and $w_{\mathrm{CT}}$ values were 1 and 0.4, respectively. By leveraging the optimal conditions, we determined the DWPC percentile threshold to recapitulate known herbal targets. Computing the DWPC percentiles for the selected herbal targets and assessing the MCC for each threshold revealed that the 40% DWPC threshold consistently yielded the highest MCC values (Fig. 4D). These findings suggest that the herbal mechanisms can be identified more accurately by carefully considering the weight of the PC.

### Evaluating the predictive performance of network models in therapeutic effects

**Fig. 5:**
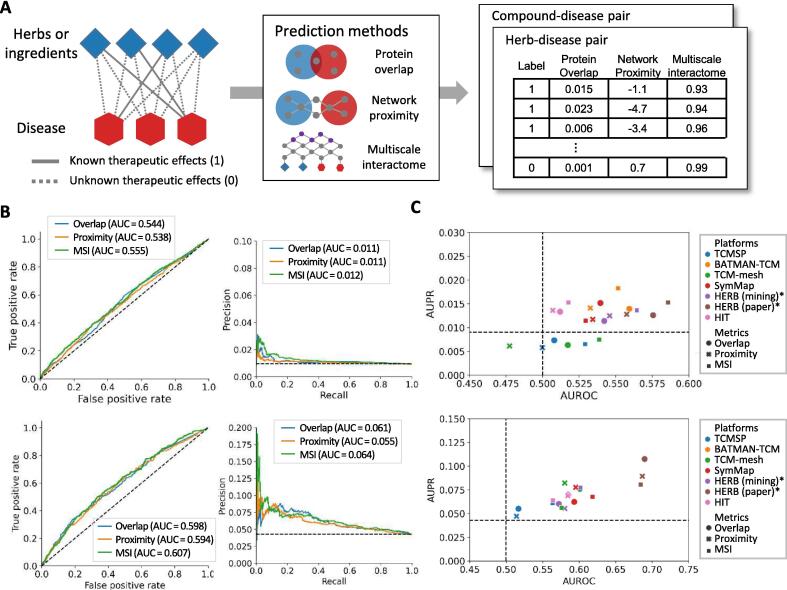
**Distribution of prediction performance for herbal compounds (upper figure) and herbs (lower figure) based on databases and prediction methods**. (**A**) Schematic representation of the tasks for determining therapeutic effects and evaluations of network-based prediction methods. (**B**) Performance curves of network-based prediction methods for known therapeutic effects. **(C**) Distribution by platform and prediction method for known therapeutic effects. Overlap: protein overlap; proximity: network proximity; MSI: multiscale interactome. HERB (mining) represents data integrated from other databases, while HERB (paper) includes experimentally validated targets. Both datasets are provided by the HERB database.

We further evaluated the utility of various network-based approaches to identify the therapeutic effects of herbal medicines. Three commonly used or potentially applicable methods, protein overlap, network proximity, and multiscale interactome, were considered. Using these methods, we calculated scores for all possible pairs of herbal ingredients and diseases, as well as those for herbs and diseases (Fig. 5A). We then performed a discrimination task using these scores to distinguish between the known and unknown associations. Notably, similar to our previous tasks, the density of known disease associations was quite low; therefore, the performance in this context might have been underestimated compared with real-world scenarios. Nonetheless, this approach was valuable for comparing the characteristics of each prediction method.

We found that all network-based approaches outperformed chance level performances for both herbal ingredient-disease and herb-disease pairs (Fig. 5B). The protein overlap method yielded satisfactory AUROC and AUPR values. However, using this approach, only approximately one-third of the herbal medicine-disease pairs yielded similarity scores greater than zero, leaving the majority of pairs unprioritized owing to a score of zero. This suggests that the protein overlap method may be limited in scope for discerning the therapeutic effects of herbal medicines and their ingredients. We also observed that the network proximity method produced lower AUROC and AUPR values compared to the other methods, suggesting that considering only closeness within a simple PPI network may not be sufficient for discerning therapeutic effects. In contrast, the multiscale interactome method outperformed the other methods both AUROC and AUPR. This highlights the importance of considering PPIs and their relationships with biological functions when determining the therapeutic effects of herbal medicines.

We analyzed performance distribution based on a combination of platforms and prediction methods (Fig. 5C). In most combinations, we confirmed that the performance surpassed chance level. Using known targets led to superior predictive outcomes for both the herbs and herbal ingredients. This suggests that, when sufficient data are available, relying solely on validated information is beneficial. However, the other methods exhibited substantial performance. HERB–multiscale interactome combination yielded the best performance for herbal ingredients, whereas SymMap–multiscale interactome combination was the most effective for herbs. Overall, these findings underscore that the predictive accuracy of the therapeutic effects of herbal medicines can vary based on the combination of network methods employed.

### Case study on determining the therapeutic effects of herbs

We sought to validate whether the identified optimal datasets and conditions could help predicting the therapeutic effects of herbs in various diseases. Using the multiscale interactome approach on the paper target, which showed the highest performance in predicting therapeutic effects, we prioritized the relationship between herbs and diseases. We discovered that nearly half (4 out of 10) of the prioritized herb-disease associations had already been reported (Table 2)). Furthermore, we explored whether the unreported associations among the prioritized results could indicate the potential therapeutic effects of the herbs. We chose to focus on prostate neoplasm because it is both a top-predicted result and a disease of significant therapeutic importance, with a rising prevalence and global impact. Given its relevance, we explored the potential treatments and mechanisms of prostate cancer in detail. We confirmed that PR and LE showed high correlation scores with prostate cancer, along with GF, which is included in the top 10 rankings, without any reported evidence. Despite their high rankings, these components have not yet been reported in literature, indicating a unique opportunity to uncover new therapeutic effects and mechanisms.

**Table 2:** Prioritized herb-indication pairs based on known herb targets and their reported evidence.

Herb ID	Disease ID	Herb name (latin name)	Disease name	Reported evidence (PMID)
herb007244	HBDIS001721	*Petiolus trachycarpi*	Acute Promyelocytic Leukemia	.
herb000960	HBDIS005061	Vinegar	Cocaine-Related Disorders	.
herb003658	HBDIS002540	*Herba ephedrae*	Pulmonary Fibrosis	21,565,143
herb003354	HBDIS002687	*Melia azedarach*	Schizophrenia	.
herb000960	HBDIS002070	Vinegar	Neoplasm Metastasis	.
herb003658	HBDIS000265	*Herba ephedrae*	Asthma	36,215,828
herb005069	HBDIS002488	*Granati fructus*	Prostatic Neoplasms	.
herb005017	HBDIS002028	*Rhizoma zingiberis recens*	Myocarditis	33,628,715
herb000960	HBDIS001615	Vinegar	Kidney Calculi	31,202,812
herb001239	HBDIS000372	*Erigeron breviscapus*	Bone neoplasms	

**Fig. 6:**
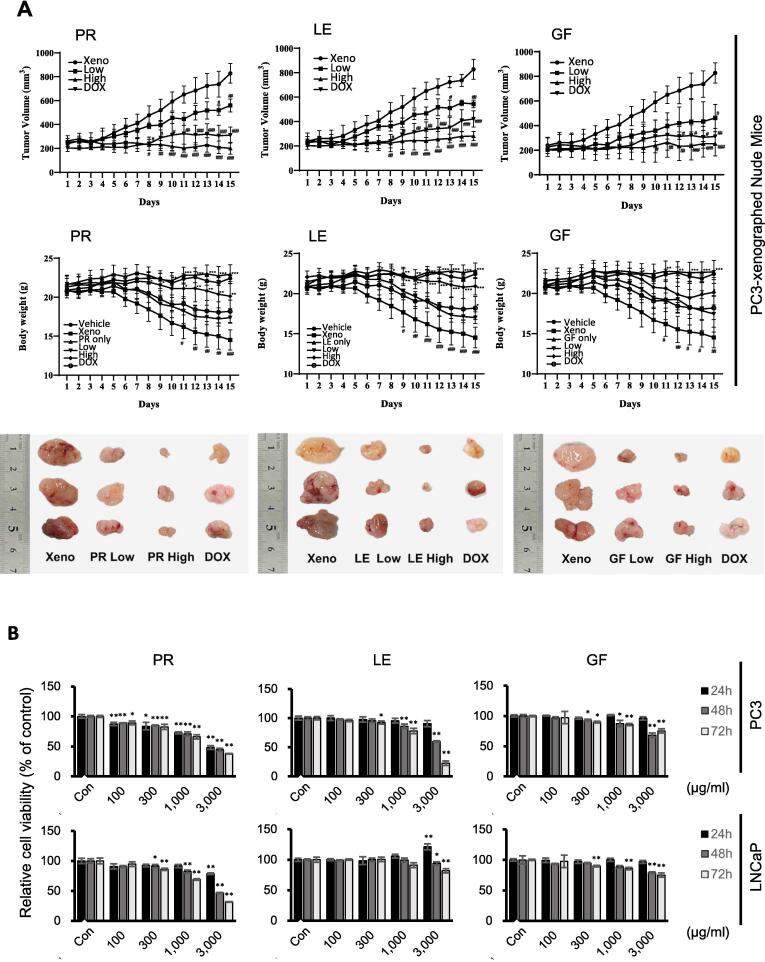
**Effects of predicted herbs on tumor growth in prostate cancers *in vivo* and *in vitro.* A.** Effects of on tumor size in PC-3 xenograft mouse model. Tumor growth (upper panel), body weight changes (middle panel) and the representation of xenograft tumor size reduction (lower panel) in a PC-3 xenograft mouse model. Tumor volumes and body weights were measured daily. Both low doses (500 mg/kg) and high doses (1500 mg/kg) of PR, LE, and GF suppressed tumor growth and reduced body weight loss (n > 8). Statistical analysis was performed using Two-way ANOVA with Dunnett’s multiple comparison test (*p < 0.05, **p < 0.01 vs. control; #*p* < 0.05, ##*p* < 0.01 vs. xenograft). PR, *Puerariae Radix*; LE, *Lithospermum Erythrorhizon*; GF, *Granati Fructus*; Xeno, xenograft control; DOX, doxorubicin. **B.** Cell viability measured using MTT assay in PC3 and LNCaP cells treated with PR, LE, and GF at the indicated concentrations and times (comparison with control, * *p* < 0.05, ** *p* < 0.01).

To assess the *in vivo* anticancer efficacy of herbal extracts, we used a PC3 xenograft mouse model (Fig. 6A). Tumor volumes and body weights were measured daily to evaluate the effects of herbal extracts on tumor growth and overall health. We found that high doses of PR, LE, and GF significantly suppressed tumor growth compared to the control group (Fig. 6A, upper panel). Tumor volumes were measured daily using a digital caliper, and the data showed a clear reduction in tumor size in mice treated with high doses of the herbal extracts. Fig. 6A (lower panel) shows representative images of tumors from each treatment group, demonstrating visual differences in tumor size. Body weight changes were monitored daily and expressed as percentages relative to day 1 (Fig. 6A, middle panel). High doses of PR, LE, and GF mitigated body weight loss typically associated with tumor progression. These results suggest that the herbal extracts not only inhibited tumor growth but also supported overall health and body weight in treated mice. Statistical analysis confirmed significant differences between treatment groups. These findings highlight the therapeutic potential of PR, LE, and GF in treating prostate cancer, as they significantly reduced tumor growth and supported body weight in a PC3 xenograft mouse model after oral administration.

**Fig. 7:**
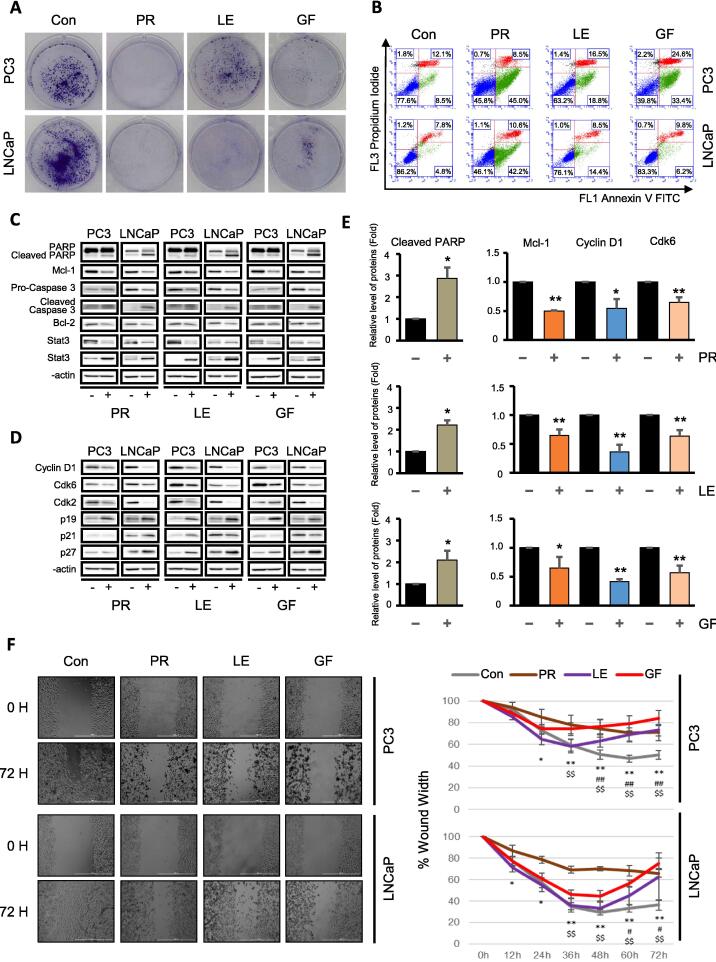
**The effects of predicted herbs on cell proliferation, migration and apoptosis in PC3 and LNCaP cells. A.** Colony formation assay: Cells treated with PR, LE, and GF at 3,000 μg/ml for 72 h were stained with Annexin V/PI to categorize the cell population into viable, apoptotic, late apoptotic, and necrotic cells (The representative bands are the results of experiments repeated at least four times). **B.** Flow cytometry analysis: Cells treated with PR, LE, and GF at 3,000 μg/ml for 72 h were stained with Annexin V/PI to categorize the cell population into viable, apoptotic, late apoptotic, and necrotic cells. **C.** Immunoblotting analysis of apoptosis-related proteins in cells treated with PR, LE, and GF at 3,000 μg/ml for 72 h. **D.** Immunoblotting analysis of cell cycle-related proteins in cells treated with PR, LE, and GF at 3,000 μg/ml for 72 h. **E.** Statistical analysis of protein of interest. (*p < 0.05, **p < 0.01 vs. control). **F.** Scratch wound healing assay: Cells were treated with PR, LE, and GF at 3,000 μg/ml for 72 h, and real-time imaging of scratch confluence was conducted (scale bar = 1,000 μm). The data represent the percentage change in scratch gap (*p < 0.05, **p < 0.01 PR; #*p* < 0.05, ##*p* < 0.01 LE; *p* < 0.01 GF vs. control). PR, *Puerariae Radix*; LE, *Lithospermum Erythrorhizon*; GF, *Granati Fructus.*

To verify the *in vitro* anticancer effects of PR, LE, and GF on prostate cancer cell lines, we performed a series of assays. The MTT assay revealed a dose- and time-dependent decrease in cell viability in PC3 and LNCaP cells, most notably at 72 h and 3,000 μg/mL (Fig. 6B). A colony-forming assay showed that treatment with these herbs inhibited clonogenic growth, indicating their long-term effects on cell survival (Fig. 7A). Flow cytometry with Annexin V/PI staining demonstrated that PR, LE, and GF significantly increased apoptosis rates in PC3 and LNCaP cells compared to controls (Fig. 7B). Western blot analysis confirmed these findings, showing that the expression of anti-apoptotic proteins Bcl-2 and Mcl-1 decreased, while markers of apoptosis, such as cleaved PARP and the β form of STAT3, increased after treatment with PR, LE, and GF. Notably, the cleaved form of caspase 3, a key executor of apoptosis, was upregulated, especially in LNCaP cells treated with these herbs (Fig. 7C and E). These assays collectively demonstrated the multifaceted anticancer activity of PR, LE, and GF, showcasing their ability to reduce cell viability, inhibit proliferation, and induce significant apoptosis.

We further explored the effects of PR, LE, and GF on cell cycle progression and metastasis. Western blot analysis indicated that PR and LE significantly downregulated the expression of Cyclin D1 and Cdk6 and inhibited Cdk2 expression, whereas GF had similar effects with specificity for LNCaP cells. Upregulation of Cdk inhibitors p19, p21, and p27 by these herbs further highlighted their influence on cell cycle regulation (Fig. 7D and E). Additionally, a wound healing assay demonstrated that PR, LE, and GF effectively hindered cell migration in both PC3 and LNCaP cells, with PR showing a notable reduction in migration as early as 12 h in LNCaP cells and 24 h in PC3 cells (Fig. 7F). Overall, our results indicate that the potent anticancer properties of PR, LE, and GF are mediated by their effects on cell migration and modulation of cell cycle-related proteins.

## Discussion

Our critical evaluation serves as a cornerstone for understanding current NP analysis of herbal medicine, focusing on underlying mechanisms and therapeutic effects. Initially, we outlined the network construction process, highlighting the diverse DBs used for compound identification, target prediction, and key analytical tools to discern herbal mechanisms and therapeutic roles. Our exploratory analysis revealed high consistency in ingredient information across herbal medicine DBs, yet significant disparities in target information, emphasizing the need for a thorough evaluation. In our recapitulation task, the performance and coverage patterns were significantly influenced by the choice of NP-DBs, their combination methods, and analysis techniques such as network centrality and weighted PC. Further analysis of the therapeutic effects highlighted the multiscale interactome method as particularly effective, especially when combined with data sources such as HERB and SymMap. Our case study on prostate cancer reinforced these findings, confirming the potential of our approach to uncover novel therapeutic effects in real-world scenarios.

Our analysis focuses on the consistency of NP-DB data, revealing significant inconsistencies, particularly in ingredient–target associations (Fig. 2). While herb–ingredient associations were generally consistent, with recall rates exceeding 90% across most database pairs except those involving HIT, ingredient–target associations showed marked divergence between databases. HIT contained very few ingredients per herb (an average of fewer than five) because it only includes ingredients with experimentally validated targets. These discrepancies likely stem from the intrinsic characteristics and data curation methods of each database. For example, SymMap and HERB compile comprehensive herb information by incorporating data from existing NP-DBs, whereas BATMAN-TCM and TCM-Mesh select ingredients based on criteria such as the availability of PubChem CIDs. In contrast, HIT focuses solely on experimentally validated targets, resulting in a much smaller dataset, with an average of fewer than five targets per herb. This inconsistency poses challenges for researchers, leading to varying interpretations of an herb's mechanism of action depending on the database. Our findings underscore the urgent need for a standardized approach to data collection and integration in NP-DBs to improve the reliability of network pharmacology analyses involving herbal medicines.

We compared various methods for key target identification in network analysis and recognized that the characteristics of these methods, according to the assumptions on which they rely, affect their applicability (Fig. 4). For instance, approaches like protein–protein interaction (PPI) network analysis, which perform effectively in social networks [41], [42], assume that targets centrally located in the PPI subnetwork are naturally pivotal. However, this assumption may not hold true in the complex networks of herbal compounds and their targets, making the efficacy of identifying key herbal targets elusive. Recent studies have highlighted that the influence of drugs is more local than global within a network [23], suggesting that the closeness between drug targets and disease-related proteins is crucial in determining therapeutic effects. Therefore, beyond global centrality measures, detailed investigation into the local relationships between herbal medicines and diseases within the network is warranted. Similarly, the use of path counts (PCs) can be limited by the uniform consideration of all paths, regardless of whether they originate from common ingredients or those with extensive target profiles. High-degree nodes often correspond to biologically general or nonspecific entities, providing limited specific information [22]. Results from the DWPC algorithm suggest that downweighting herb-compound and compound-target paths involving high-degree nodes can elucidate known mechanisms of herbal medicines more accurately. Differentiating the weights of PCs based on node characteristics—such as downweighting paths associated with high-degree nodes—could refine the analysis. Adjusting path weighting in this way may lead to a more accurate elucidation of key mechanisms, enhancing our understanding of how herbal medicines exert their therapeutic effects. Overall, these insights highlight the need for nuanced analytical techniques that consider the unique properties of herbal medicine networks to improve key target identification.

To the best of our knowledge, this study represents a pioneering effort to systematically investigate the therapeutic effects of natural products (Fig. 5). Historically, the identification of disease treatments through NP has predominantly focused on the measures of protein overlap and network proximity. These are conducted based on the assumption that a drug that tends to overlap with a disease and its proteins will treat that disease, or that a target occupying an important network position in the relationship between selected proteins will play a crucial role. However, the applicability of these foundational beliefs to understand the intricacies of natural products remains a topic of debate. Our findings suggest that protein overlap is limited and only effective for a restricted spectrum of therapeutic effects. Although network proximity encompasses protein relationships within a network, its efficacy often mirrors random outcomes, suggesting that merely focusing on protein interactions in natural products may generate misleading conclusions. In contrast, our research indicated that evaluating the effects within a multiscale interactome can provide a more reliable prediction for identifying therapeutic effects.

To validate the potential of a novel therapeutic approach for prostate cancer predicted by our research, we conducted *in vitro* experiments to confirm its effects. In our study, we confirmed that the predicted herbs PR, LE, and GF inhibited cell viability, proliferation, and migration by regulating proteins involved in apoptosis and cell cycle regulation (Fig. 6). Moreover, we confirmed the *in vivo* effects of the predicted herbs on tumor growth inhibition in nude mice xenografted with PC3 cells (data not shown). PR is a widely used traditional herb for various conditions such as cardiovascular diseases, diabetes mellitus, and deafness [43]. Although studies on the anticancer effects of PR *in vivo* and *in vitro* are lacking, the isoflavones present in PR induce apoptosis and cell cycle arrest in the G2/M phase in breast cancer cells [44]. LE has antioxidant properties [45] and suppresses high-fat diet-induced obesity [46]. In addition, LE has also been shown to exhibit anticancer effects by inducing apoptosis and G1 phase arrest in B16F10 melanoma cells. Additionally, it has shown potential anticancer effects in a C57BL/6 mouse model [47]. GF has demonstrated anti-inflammatory effects both *in vivo* and *in vitro* [48]. It has therapeutic and preventive effects against various chronic human diseases, including an atherogenic lipoprotein profile, imbalanced antioxidative status, and disrupted glucose tolerance [49]. In line with this, several ingredients have been studied as anti-cancer agents in clinical trials [50]. Dietary supplements like saw palmetto and green tea extract have also shown effects on prostate cancer in randomized controlled trials (RCTs) [51]. Notably, clinical studies have explored herbal components such as genistein and daidzein found in PR, as well as vitamin C and catechin present in GF, for their roles in regulating prostate cancer progression [52], [53], [54]. These previous and current findings suggest that the predicted herbs in this study have potential efficacy against prostate cancer. However, further research, including RCTs, is needed to confirm the beneficial effects of PR, LE, and GF on prostate cancer and other human cancers.

This study may contain several limitations that may require further evaluation and refinement. First, the known therapeutic effects analyzed are primarily based on *in vivo* experiments, suggesting the need for additional clinical studies to validate these findings in a real-world context. Second, our analysis relied on a single database for known target and therapeutic effects, which may introduce bias due to the limited scope and curation criteria of the database. Lastly, while our computational methods are robust, they do not fully account for the complex pharmacokinetics and interactions of multiple compounds within herbal formulations, which could influence therapeutic outcomes. Nevertheless, this study represents the first systematic analysis and validation of the therapeutic effects of herbal medicine through network pharmacology, laying a strong foundation for future research in this area.

## Conclusion

Our study systematically evaluated the methodologies of NP in herbal medicine and provided essential insights into the mechanisms and therapeutic effects of herbal ingredients. We emphasize the significance of methodological approaches in network construction and analysis, particularly the advantages of multiscale interactomes over traditional methods, such as protein overlap and network proximity. Our empirical validation further affirmed the potential of NP to identify and substantiate novel therapeutic effects, as exemplified by its successful application in prostate cancer treatment, thereby establishing a precedent for future research in this promising field.

## CRediT authorship contribution statement

**Won-Yung Lee:** Conceptualization, Methodology, Writing – original draft, Supervision. **Kwang-Il Park:** Investigation, Visualization, Writing – original draft. **Seon-Been Bak:** Software, Formal analysis, Writing – original draft. **Seungho Lee:** Formal analysis, Project administration, Supervision, Writing – review & editing. **Su-Jin Bae:** Validation, Resources. **Min-Jin Kim:** Formal analysis, Data curation. **Sun-Dong Park:** Resources, Writing – review & editing. **Choon Ok Kim:** Resources, Writing – review & editing. **Ji-Hwan Kim:** Funding acquisition, Writing – review & editing. **Young Woo Kim:** Conceptualization, Supervision, Writing – review & editing, Funding acquisition. **Chang-Eop Kim:** Conceptualization, Methodology, Writing – review & editing.

## Funding

This research was supported by grants from the National Research Foundation of Korea (NRF) funded by the Korean government (MSIT) (grant numbers RS-2023–00243363, RS-2023–00218419, RS-2024–00449029, and RS-2024–00335030), the Ministry of Education (NRF–2022R1I1A2066653 and 2022R1I1A3053818), and the Korea Health Technology R&D Project through the Korea Health Industry Development Institute (KHIDI), funded by the Ministry of Health & Welfare, Republic of Korea (grant number: HF20C0212).

## Declaration of competing interest

The authors declare that they have no known competing financial interests or personal relationships that could have appeared to influence the work reported in this paper.
