# Supplementary material for: Evaluating current status of network pharmacology for herbal medicine focusing on identifying mechanisms and therapeutic effects
Source: J Adv Res. 2024 Dec 25;76:799–815. doi: 10.1016/j.jare.2024.12.040 (PMC12793800; doi:10.1016/j.jare.2024.12.040)
Supplement: Supplementary Data 1 [file mmc1.pptx]

## Slide 1
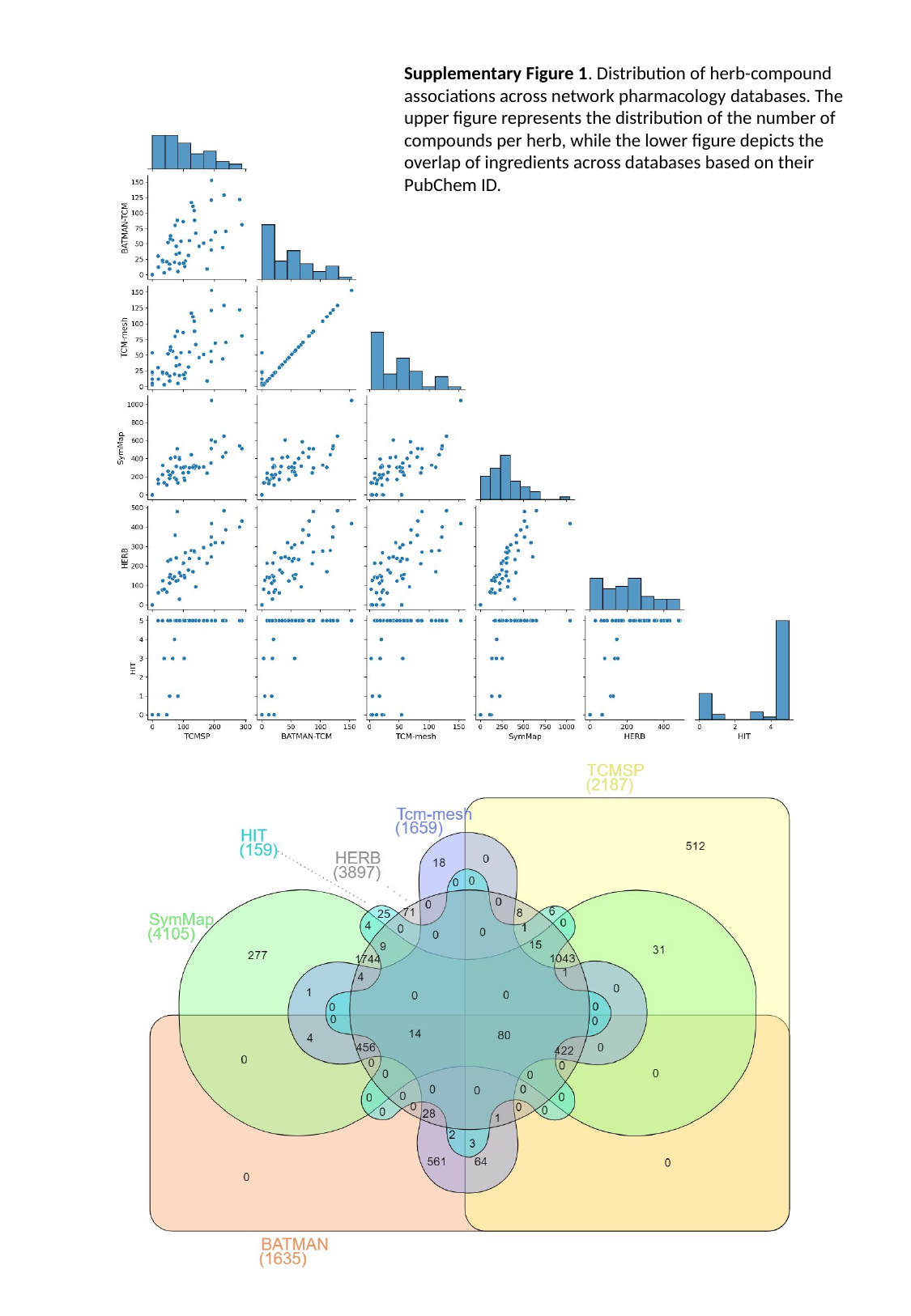

Supplementary Figure 1. Distribution of herb-compound associations across network pharmacology databases. The upper figure represents the distribution of the number of compounds per herb, while the lower figure depicts the overlap of ingredients across databases based on their PubChem ID.

## Slide 2
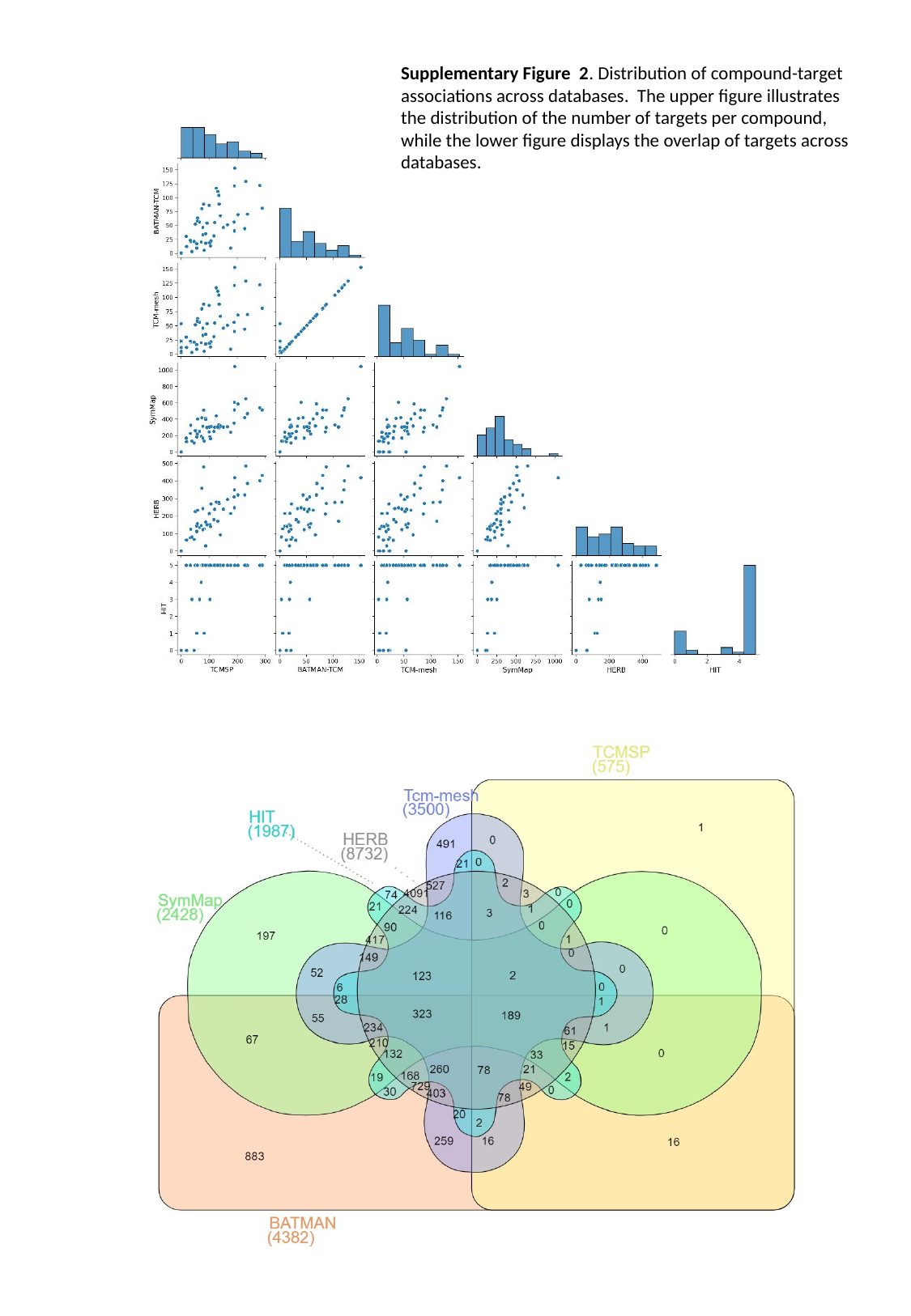

Supplementary Figure 2. Distribution of compound-target associations across databases. The upper figure illustrates the distribution of the number of targets per compound, while the lower figure displays the overlap of targets across databases.

## Slide 3
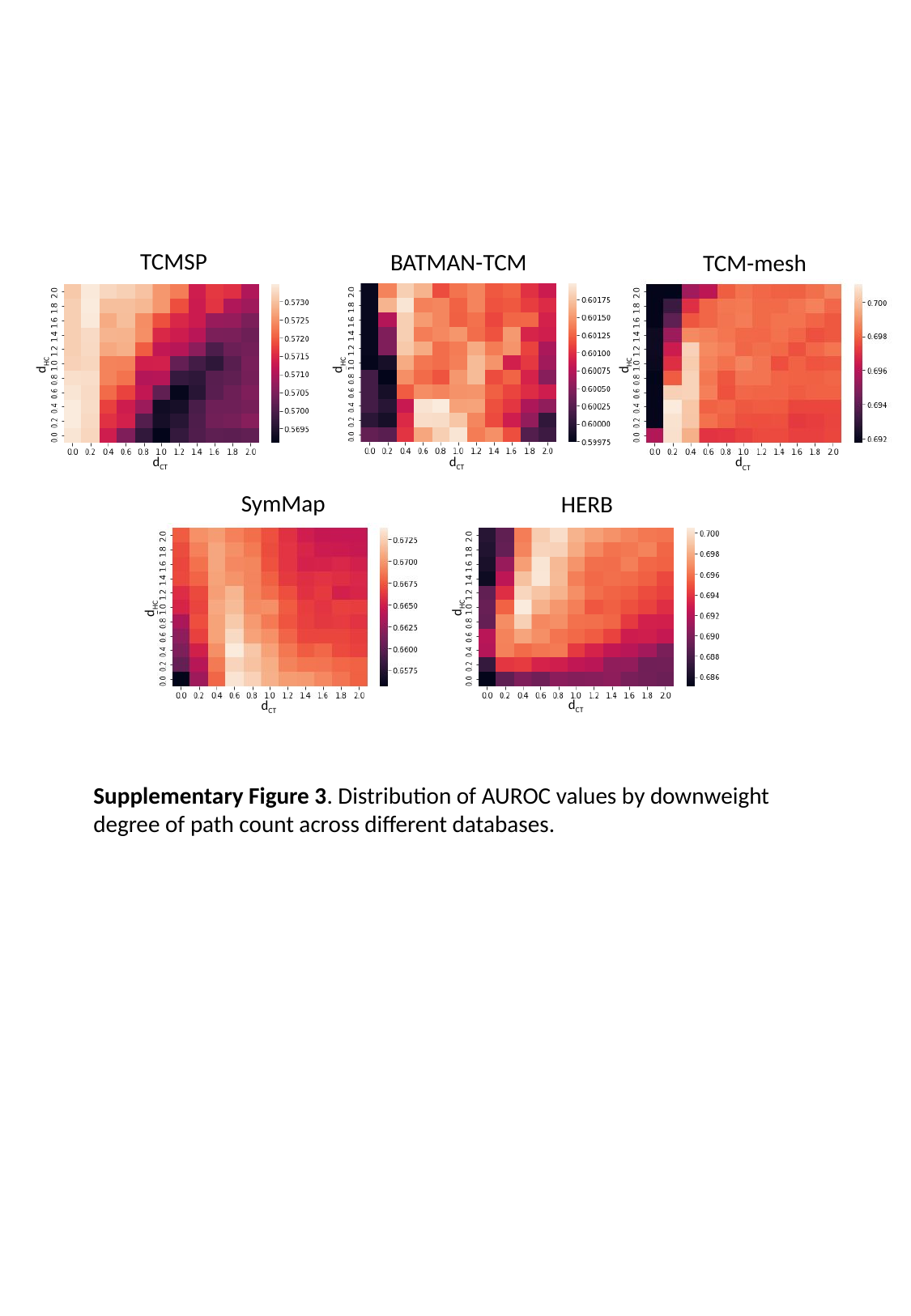

TCMSP
BATMAN-TCM
TCM-mesh
dHC
dHC
dHC
dCT
dCT
dCT
SymMap
HERB
dHC
dHC
dCT
dCT
Supplementary Figure 3. Distribution of AUROC values by downweight degree of path count across different databases.
